# Water entropy at the threonine-rich surface of antifreeze and ice-nucleating proteins: small changes make a big difference†

**DOI:** 10.1039/d4sc08383k

**Published:** 2025-05-09

**Authors:** Deabsis Saha, Rahul Aich, Arnab Mukherjee, Biman Jana

**Affiliations:** a College of Integrated Science and Arts, Arizona State University Mesa Arizona 85212 USA; b School of Chemical Sciences, Indian Association for the Cultivation of Science Jadavpur Kolkata 700032 India pcbj@iacs.res.in; c Department of Chemistry, Indian Institute of Science Education and Research Pune 411008 India arnab.mukherjee@iiserpune.ac.in

## Abstract

Heterogeneous ice nucleation is vital for various natural processes and for maintaining global sea levels. Although different ice nucleating proteins (INPs) have been discovered on naturally formed ice, many organisms possess antifreeze proteins (AFPs) which are structurally similar to INPs and yet prevent ice growth in their body fluid. In this study, we investigate the ice nucleation efficiency of INPs over AFPs by looking into water entropy near these proteins. Using all-atom molecular dynamics simulations and a method to calculate the entropy of individual water molecules, we found distinct water entropy patterns near AFPs and INPs. For the INP structure, water molecules hydrogen-bonded to threonine residues on the ice-binding surface (IBS) exhibited the most significant entropy decrease, likely lowering the entropic barrier for ice nucleation. Even for water near the IBS of the two AFPs studied, the entropy patterns have been found to be dissimilar. Our analysis reveals that the entropy patterns stem from varying fluctuation levels of threonine side chains on the IBS. Consequently, for two INP conformations differing in the orientation of a conserved loop near the IBS, one structure has been found not to lower water entropy as effectively as the other. Our study reveals that larger surface areas or continuous threonine patches are not the only criteria that create differences between AFPs and INPs. The extent of rigidity and consequently the change in water entropy behavior enhances the ice nucleation efficiency of INPs under moderate supercooling conditions.

## Abstract 2

Low-entropy water near threonine on ice-nucleating proteins promotes ice formation at moderate supercooling, while flexible threonine on antifreeze proteins keeps water bulk-like, preventing ice nucleation.

## Graphical abstract

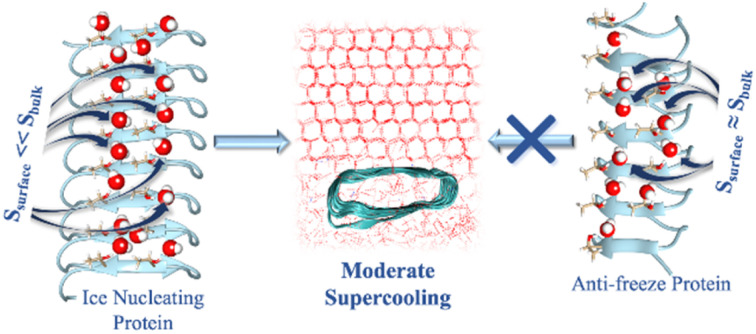

## Introduction

The essential role of ice nucleation in maintaining global sea levels and enabling cryopreservation^1^ necessitates a detailed exploration of how ice-nucleating proteins (INPs) effectively promote ice formation. The kinetic barrier associated with homogeneous nucleation of ice keeps water in a liquid state down to −35 °C.^2^ Consequently, it has been suggested that ice formation above −20 °C can occur only through heterogeneous ice nucleation (HIN).^3^ Among different ice nucleating agents, bacterial ice-nucleation activity is of particular interest, not only because these bacteria cause frost damage^4^ but also due to their presence in ice, hail, and snow, indicating their role in atmospheric ice nucleation.^5,6^ The origin of bacterial HIN activity stems from certain cell-membrane bound INPs, which are believed to facilitate crossing of the nucleation barrier required for ice formation by arranging water molecules into an “ice-like” structure.^7–9^ As a result, these INPs can promote ice nucleation at temperatures as warm as −2 °C, leading to disruptions in the cell membrane.^10^

Alternatively, another class of proteins known as antifreeze proteins (AFPs) plays a crucial role in the survival of organisms in sub-zero environments. Despite the structural diversity of AFPs found in organisms such as insects, fish, plants, and bacteria,^11–13^ these proteins are believed to protect against freezing by adsorbing to the surface of ice crystals *via* their ice-binding surface (IBS) through a common mechanism called the adsorption-inhibition mechanism.^14^ This adsorption leads to curvature formation on the ice surface between bound AFPs, lowering the freezing temperature in a noncolligative manner.^12^ While AFPs function in opposition to INPs, there are notable similarities between the two. The ice-nucleating efficiency of INPs also relies on their ability to bind to ice crystals, similar to AFPs.^15^ Interestingly, AFPs have also shown evidence of ice nucleation,^16^ though in a size-dependent manner.^17^ This shared behaviour is thought to originate from the TxT repeats found in the water-exposed α-helix regions of several INPs and AFPs,^18–21^ where T stands for threonine and x represents a non-conserved amino acid.^22–24^ In this arrangement, the distance between the threonine hydroxyl groups aligns with the spacing of water molecules on the prismatic and basal planes of ice,^22–24^ providing a template for ice recognition on these surfaces.^25^ Although there is evidence suggesting the presence of “ice-like” water on the surface of AFPs,^26^ their ice-nucleating ability is significantly lower than that of INPs.^16,25,27^ The larger area of the IBS on INPs compared to AFPs has been the primary explanation for this difference.^28,29^ Similar size dependence of the ice nucleating surface has been observed for non-biological surfaces as well.^30^ However, the behavior of water molecules at the IBS of both INPs and AFPs remains to be fully understood.

For any ice nucleating agent, the interaction between the interfacial water and the surface is critical.^31,32^ The fact that it is possible to accurately predict a material's ice nucleating efficiency from the nature of the first layer of water near a surface^33^ indicates that a closer look at the water behavior at lower temperatures near AFPs and INPs can reveal the intricate interaction patterns that govern the ice nucleation at the INP surface. Water shows numerous anomalies when supercooled.^34,35^ One of the reasons for such anomalies has been attributed to the hydrogen bonding (H-bond) properties of water, which shows a non-Arrhenius behavior for H-bond lifetimes when supercooled.^36^ Such properties provide an indication of enthalpic stabilization of water at lower temperatures. Therefore, the barrier for water to ice formation is likely to come from water entropy. Also, the entropy of water has been shown to play a crucial role in the formation of metastable stacking-disordered ice,^37,38^ which eventually converts to a more stable hexagonal ice form. Therefore, a closer look at water entropy near AFPs and INPs can reveal whether the activity of INPs is solely due to their larger size compared to AFPs.

In this study, we aimed to differentiate water molecules near the IBS of AFPs and INPs in terms of their entropy calculated individually. Using all-atom molecular dynamics (MD) simulations and with a method that calculates the translational and rotational entropy of individual water molecules, we have investigated the water entropy near two different AFPs and near two truncated INP structures obtained from two different models. For comparison, we also evaluated entropy values near threonine residues on a non-ice-binding surface. Although it is well established that the larger surface area of INPs promotes the formation of more ice-like water molecules^28,29^ and the truncated INPs are less likely to be effective ice-nucleators, all-atom MD simulations of full length INPs or their aggregates remain computationally challenging. Therefore, we have focused on understanding how the INP surface facilitates ice-like ordering in water, under the assumption that surface water properties are largely preserved across larger INP surfaces. To validate this assumption, we also calculated a few properties of water near a larger INP system. The results indicate that water near the threonine residue patches at the IBS of AFPs and INPs have different entropies with water near one of the INPs having the lowest entropy values. Even for the two AFPs studied, we have found differences in their entropy pattern which are likely to influence their ability to nucleate ice under moderate supercooling conditions. We have investigated the structural features that could give rise to different water entropy patterns near the different IBSs. Our analysis indicates that a small change in the structure leads to differences in the fluctuations of threonine residues at the IBS of different systems. Such differences lead to differences in water structures and consequently their entropy patterns. These findings emphasize the fact that the differences in AFPs and INPs not only come from variation in surface area or the presence of consecutive threonine patches for the IBS, but the water at the IBS of AFPs and INPs also exhibits a microscopic difference in behavior. The results of our study provide insights into the design strategies of biological and synthetic ice nucleating agents (INAs) which could be useful in several areas of environmental significance.

## Results and discussion

### Rotational entropy of water near the IBS of AFPs and INPs has different patterns

In this study, we will discuss the rotational entropy of individual water molecules near the IBS of two different AFPs and near two different models of truncated INP structures. The AFPs studied here are the spruce budworm AFP (PDB: 1M8N)^23^ and *Tenebrio molitor* AFP (TmAFP) (PDB: 1EZG).^22^ The INP structure used here is the *P. borealis* INP reported in ref. 39 whose structure has been generated using an artificial intelligence-based program AlphaFold v2.0.^40^ Since the original structure reported is large in size, we have used a truncated section of the whole INP (INP-2022) structure in which the residues between 894 and 1025 have been included that contain eight β-sheet rich coils each having 16 residues. The other fragment of the INP (INP-old) has been obtained from AlphaFold v1.0.^41^ In this case also, we have kept eight coils and the residues included here are between 824 and 955. Although the residue numbers are different in the two systems, the selection was made in order to obtain a flat IBS for both. Additionally, they both present continuous patches of threonine residues, which makes their IBS similar for comparison. The systems have been subjected to all-atom MD simulations in which the proteins were placed in a cubic simulation box and solvated using a TIP5P water model.^42^ The details for the simulations are given in the Methods section. For the entropy calculations, 200 ns long simulations at constant volume have been carried out at 273 K temperature and 1 bar pressure with frames saved at every 0.1 ps. The trajectories thus obtained have been used for entropy calculations for individual water molecules whose method^43^ has been discussed in the Methods section. Previously, the method has been found to correctly calculate the entropy change near different ions.^44^ Therefore, the method is expected to provide qualitatively correct entropy patterns near the IBS of different systems. For each system, we have calculated entropy for 100 nearest water molecules from the C_α_ atoms of the threonine residues located at the IBS of each system. For each water molecule, 2 × 10^6^ frames from the 200 ns trajectory of all systems were analyzed. In total, nearly 500 entropy calculations were performed for water near the AFPs, INPs, and non-AFP systems considered in this study. To compare the values with pure water entropy at 273 K, simulation of pure water also has been carried out. In this case also, water entropy has been calculated by considering one of the water molecules as a reference solute.

**Fig. 1:**
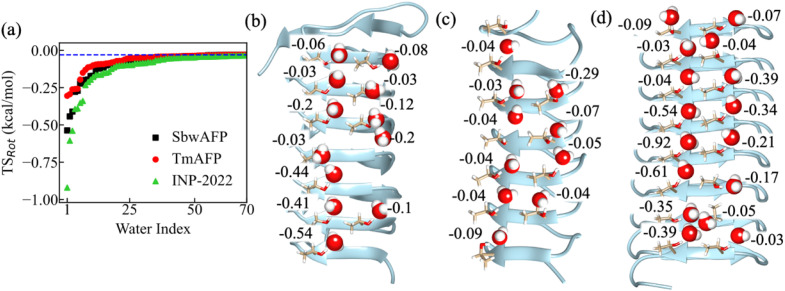
(a) The *TS*_Rot_ for water molecules near the IBS of SbwAFP, TmAFP and INP-2022 in ascending order. The blue dashed line shows the average *TS*_Rot_ of pure water molecules at 273 K. The individual *TS*_Rot_ values of water molecules H-bonded to the threonine hydroxyl groups for (b) SbwAFP, (c) TmAFP, and (d) INP-2022 are shown. The unit for entropy values (multiplied with temperature) is kcal mol^−1^.

We first compare all the rotational entropy (*TS*_Rot_, *T* being the temperature) values calculated for water molecules near the IBS of SbwAFP, TmAFP and INP-2022 and check how they behave. The *TS*_Rot_ values for the water molecules calculated near SbwAFP, TmAFP and INP-2022 are shown in Fig. 1(a) in an ascending manner. The *x*-axis, labeled as “Water Index,” represents individual water molecules ranked based on their *TS*_Rot_ values, with lower-entropy water molecules assigned lower indices and higher-entropy water molecules assigned higher indices. For the pure water system simulated at 273 K, the average *TS*_Rot_ for all 50 water molecules was found to be −0.03 kcal mol^−1^. This value is shown in Fig. 1(a) as a blue dashed line. The figure shows that water entropy reaches the pure water value after a point. The figure shows that the water molecules closest to all the protein surfaces show lower entropy compared to pure water. However, the number of low entropy water seems to be higher near the INP-2022 system, with several water molecules showing significant differences from pure water entropy. This suggests enhanced water ordering, which is likely to facilitate heterogeneous ice nucleation on the IBS of INPs. To check whether the entropy values have converged or not in our simulations, we have checked the convergence for several water molecules near the SbwAFP system. Fig. S1 in the ESI† presents the entropy values calculated at different time intervals. The plot illustrates entropy values obtained using varying trajectory lengths for seven randomly selected water molecules, each represented by a different color. The plot indicates that for water molecules having a wide range of entropy values, the 200 ns long simulation is sufficient to get converged. To estimate the error in our calculated *TS*_Rot_ values, we analyzed the first and last 100 ns of the 200 ns trajectory of the SbwAFP system. Entropy values were computed for the 30 water molecules closest to the IBS, and the associated errors were determined. The error bars, shown in Fig. S2 in the ESI,† indicate that the errors in the calculated entropy values are small, consistent with previous entropy calculations in the solvation shell of ions.^44^ The threonine residues on the IBS are often considered to contribute towards binding with growing ice faces at low temperatures.^24^ Therefore, we inspect the *TS*_Rot_ for water molecules which remain H-bonded to the threonine hydroxyl groups on the IBS. The individual values for these water molecules at the IBS are shown in Fig. 1(b)–(d) for SbwAFP, TmAFP and INP-2022, respectively.

From the figure, we find that several water molecules near SbwAFP show lower *TS*_Rot_ values compared to bulk water. For some of the water molecules, the lowering has been found to be up to 15 times the bulk water value. For the TmAFP system shown in Fig. 1(c), we see that although some of the water molecules have lower entropy compared to pure water, the values here do not decrease as much as those of the SbwAFP system. Previously, the simulation study by Hudait *et al.*^45^ reported that water entropy near TmAFP is lower than that of bulk water. It has been claimed that such low entropy indicates ordering of the surface water although there was no evidence for formation of clathrate or ice-like structures.^45^ In a different study, different models of water were used to show that the solvation shell water near the IBS of TmAFP has no ice-like or clathrate-like characteristics.^46^ From our calculation of individual water entropy, we also report slight lowering of entropy for water at the IBS of TmAFP compared to bulk water. However, the extent of entropy decrease is more significant near the SbwAFP system than near TmAFP. The thermal hysteresis data reported by Kozuch *et al.*^47^ for SbwAFP and TmAFP systems using a neural network method and through extrapolation of experimental values indicate that SbwAFP is more effective in preventing ice growth in solution compared to TmAFP. Hence, it can be argued that the thermal hysteresis patterns for different AFPs may originate from the water ordering tendency of the AFP's IBS in solution. The calculated entropy values in this study point towards a direct correlation between rotational water entropy and ice-binding efficiency for AFPs from different species.

For the INP-2022 system shown in Fig. 1(d), we find that *TS*_Rot_ values get even lower as we go towards the middle region in the eight-coil structure. In this case, a significantly larger number of water molecules can be seen to have lower *TS*_Rot_ values compared to bulk water. To compare the entropy values of water near the IBS with a system having no ice-binding characteristics, we have simulated a truncated non-AFP system (PDB ID: 2BM4) and calculated the entropy of water near threonine residues on a flat surface of this protein. The values for these water molecules are shown in Fig. S3 in the ESI.† The values mentioned in the figure show that water entropy near threonine residues on any surface will have a lower value compared to bulk water at 273 K. Such lowering is also observed in the case of TmAFP in our calculations. Note here that the values may differ for threonine residues present at a different protein surface having different characteristics. From the values observed, it can be argued that the lower entropy values observed near TmAFP are not a characteristic of its IBS. Rather, it is the effect of threonine residues that bind to its surrounding water molecules and thus lower their entropy. Such lowering becomes much more pronounced in some of the AFPs and in the case of INP systems.

The method used for calculation of entropy gives both translational (*TS*_Trans_) and rotational entropy values for individual water. Hence, the *TS*_Trans_ values for water near the IBS of different systems have been obtained. The values for SbwAFP, TmAFP and INP-2022 are shown in Fig. S4(a)–(c) in the ESI,† respectively. In this case, although we observe a reduction in entropy for INP-2022 compared to the other systems, the *TS*_Trans_ of water molecules at the center of INP-2022 is not the lowest, in contrast to the *TS*_Rot_, which shows the lowest values at the center. Additionally, the two AFPs do not show any clear difference in terms of *TS*_Trans_. Therefore, we find that the rotational entropy pattern is a more appropriate parameter to investigate the ice nucleation propensity of AFPs and INPs. Hence, we will focus our analysis on explaining the *TS*_Rot_ behavior observed in the study in the following sections of the manuscript.

The role of methyl groups on the threonine residues has also been claimed to be crucial for AFPs binding to the ice surface.^45^ Therefore, we have also investigated the *TS*_Rot_ patterns near the methyl groups of threonine residues on the IBS for TmAFP and INP-2022. The values are shown in Fig. S5 in the ESI.† From the values, it appears that for water near the TmAFP system, the values are very similar to those of bulk like water with nearly no difference in their values. For the INP-2022 system, although few of the water molecules have low entropy values, the difference in entropy patterns between the two systems is not as significant as the ones H-bonded to the hydroxyl groups. Overall, it appears that the entropy of several water molecules near INP-2022 is significantly lower compared to pure water at the same temperature. Previous studies have provided the evidence for disordered water molecules on the surface of TmAFP^46^ and the presence of ordered water near the IBS of INPs from ice nucleating *Pseudomonas syringae* bacteria at water melting temperature.^48^ Since water ordering can have direct correlation with the *TS*_Rot_ values, we can say that our calculated entropy patterns show a qualitatively correct picture of water behavior near the IBS of different systems. However, quantifying the exact impact of entropy patterns on heterogeneous ice nucleation remains challenging due to the extensive time and length scales involved, which exceed the scope of this study. Additionally, the requirement of large surface area of INPs and their aggregates for ice nucleation^28,29^ makes direct ice nucleation simulation on INP surfaces computationally prohibitive. In a previous study by our group,^25^ we observed that the arrangement of threonine hydroxyl groups closely aligns with the basal plane of hexagonal ice, likely facilitating the growth of hexagonal ice near the IBS of INPs. In a more recent study,^49^ we conducted water-to-ice nucleation simulations using enhanced sampling methods. By applying an external bias on water molecules near the IBS of AFPs, INPs, and non-INP surfaces, we found that stable ice nuclei formed most readily near the IBS of INPs with a smaller free energy cost of formation compared to other systems. This occurred despite the likely reduced ice-nucleation efficiency of the truncated INP structure due to its smaller surface area. We hypothesized that water molecules near the INP's IBS exhibit higher structural order and lower entropy, reducing the entropic barrier to ice nucleation.^49^ Through our entropy calculations of individual water molecules, we validate this hypothesis, confirming that water near the IBS of INPs indeed possesses lower entropy compared to the IBS of other systems.

To assess the enthalpic stabilization of water during ice nucleation, we performed additional simulations of SbwAFP, TmAFP, and INP-2022 embedded in ice at 273 K. To insert the proteins into the ice, we followed the ice growth simulation protocol from our previous study.^50^ Once ice formed around the IBS, each system underwent a 10 ns NPT simulation using the same parameters as those in the other simulations. When analyzing the interaction energy between the protein and surface water molecules, we found no significant difference between water and ice at 273 K. As a qualitative measure of enthalpic stabilization of ice-like water compared to bulk water near the IBS, we calculated the average number of hydrogen bonds (H-bonds) the surface water molecules form with its surroundings. We used a distance cutoff of 3.5 Å and an O–H⋯O angle threshold of 30°. Since water molecules with more H-bonds are generally in a more energetically stable state, we compared the average number of H-bonds per surface water near the IBS under normal conditions and ice-like conditions. The results for 50 closely interacting water molecules are shown in Fig. S6(a) and (b) in the ESI† for non-ice water and ice like water, respectively, for SbwAFP, TmAFP, and INP-2022 systems. As expected, water in the ice-like state exhibited a higher average number of H-bonds. However, the values across all systems were similar in water (Fig. S6(a)†) and in ice (Fig. S6(b)†) with only slight exception for TmAFP which shows fewer H-bonds compared to the others when in ice, suggesting that the enthalpic gain upon ice formation is comparable. However, our previous study^49^ found that the free energy barrier for ice nucleation is smaller near INPs compared to the others. This implies that the primary barrier for water-to-ice transition arises from entropy. Since water to ice formation is likely to result in rotational constraints on water molecules, lowering of *TS*_Rot_ near the INP structure indicates its ability to make its solvation shell water more ice like. Such ice like water is likely to initiate ice nucleation on the surface of INPs even at moderately low temperatures.

Our calculations also explain why some AFPs show ice-nucleating efficiency, albeit to a lesser extent than INPs. The *TS*_Rot_ values for several water molecules near SbwAFP are found to be lower than those for bulk water. However, in this case, we see that the threonine ladder is not continuous on one side. Hence, only a few water molecules will undergo lowering of entropy which could contribute to the lower effectiveness of such AFPs towards ice nucleation. This explains why a partially purified 164 kDa AFP structure could show ice nucleation activity while a shorter 72 kDa fragment did not have such activity.^17^ Additionally, our results indicate that the difference in ice nucleation activity for INPs and AFPs may not only originate due to the larger size of INPs as argued in several studies.^16,29^ Different AFPs modulate water properties differently and hence, even a larger size of such AFPs may not be sufficient to effectively nucleate ice under moderate supercooling conditions. Other factors may also be crucial for determination of the efficiency of a surface in terms of ice nucleation. In the next section, we will investigate the structural difference between water molecules near the IBS of AFPs and INPs.

### Structural and dynamic patterns near the IBS of AFPs and INPs are different

**Fig. 2:**
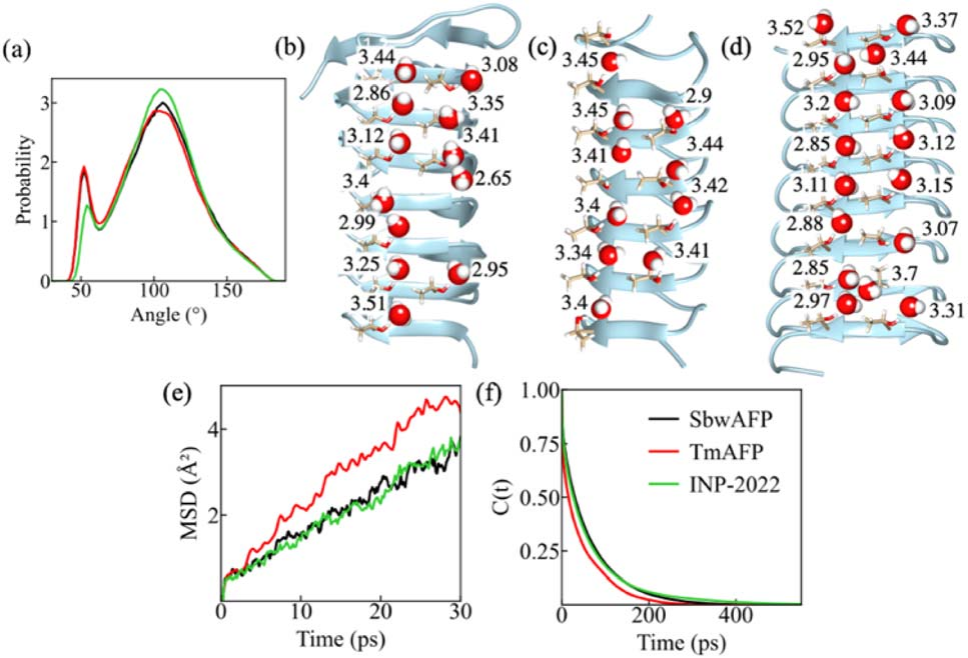
The distribution of the O–O–O-angle for water in the vicinity of threonine residues on the IBS of SbwAFP, TmAFP and INP-2022 (a). The average number of H-bonds per water for individual water molecules at the IBS of (b). SbwAFP, (c) TmAFP, and (d) INP-2022. The mean square displacement of water molecules near the IBS (e) and the decay of the correlation function *C*(*t*) with time for water molecules H-bonded to threonine residues on the IBS (f) of SbwAFP (black line), TmAFP (red line) and INP-2022 (green line).

Subtle differences in the structure for solvation shell water around the IBS can result in variation of water entropy values. Therefore, to show the structural difference for water near the IBS of AFPs and INP-2022, we first measured the O–O–O angle distribution for water on the IBS. For this purpose, the water molecules, closest to each threonine oxygen atom of the IBS, are selected first. Then two water molecules within 3.5 Å are considered from the selected (central) water molecules. The angle between two vectors connecting the water oxygen atoms from the central oxygen is calculated. This angle parameter gives useful information about the tetrahedral arrangement of water molecules and is shown in Fig. 2(a). The perfect tetrahedral ordering of water indicated by a peak at around 109° is most evident at the IBS of INP-2022 and least for TmAFP. The permutation reduction method used to calculate the *TS*_Rot_ values can be used to calculate different structural properties of individual water molecules near the IBS. Therefore, we investigate the H-bonding behavior and tetrahedral order of water molecules near the IBS threonine residues in order to check whether the entropic behavior correlates with the water structure or not. The significance of H-bonding between the threonine hydroxyl groups and surface water at the IBS has been well documented through experiments^48^ and simulations.^51^ In our calculations, we determine the number of H-bonds per water by considering a distance cutoff of 3.5 Å from the reference water oxygen atom to the oxygen atoms from a threonine residue or another water molecule followed by a donor–hydrogen–acceptor (O–H⋯O) angle within 30°. For pure water, the average number of H-bonds was first calculated for 30 permuted water molecules and the value has been found to be 3.6 per water molecule at 273 K. The average number of H-bonds for water which are H-bonded to the hydroxyl group of threonine residues on the IBS of SbwAFP, TmAFP and INP-2022 are shown in Fig. 2(b)–(d). Note that the value obtained here for the water H-bond number is dependent on the water model used.

The figures show that the number of H-bonds per water is lowest for water around the IBS of INP-2022 among the three systems. Even for the two AFPs, SbwAFP shows a slightly lower number of average H-bonds compared to TmAFP. Since a higher number of H-bonds per water is an indication of bulk like water, the water around TmAFP appears more similar to bulk water. Similar patterns have been observed for water tetrahedral parameters as well. As the water near protein surface may not always have four neighboring oxygen atoms within a H-bonding donor–acceptor distance of 3.5 Å, we have used the conditional tetrahedral order parameter (*t*_h_),^52^ which is more appropriate for systems where the central water molecule can have 2 to 4 neighbors. Previously, *t*_h_ has been shown to indicate enhancement in tetrahedrality for water in the first solvation shell of the Maxi protein, a naturally occurring homodimeric nanopore, compared to bulk water,^53^ although the values differed only slightly. In our case, the *t*_h_ has been obtained for water molecules H-bonded to threonine residues on the IBS of AFPs and INP-2022. The distribution of *t*_h_ for all the water molecules is shown in Fig. S7,† followed by the *t*_h_ values for individual water molecules in Fig. S8 in the ESI† for water molecules considered near SbwAFP, TmAFP and INP-2022. From the distribution shown in Fig. S5,† it appears that the major peak in the distribution for all the systems appears at the same position. However, for water near INP-2022, the probability of *t*_h_ at higher values appears to be slightly more than that of the other systems. Similarly, *t*_h_ values for individual water molecules also show a slight increase near the INP-2022 system compared to the others, with the TmAFP system having the lowest values. These observations also explain why SbwAFP has better antifreeze ability compared to TmAFP. The H-bonding behavior, along with higher tetrahedral order near INP-2022 indicates that water thermodynamics near INP-2022 is different not only in terms of entropy, but also in enthalpy since the lack of H-bonding would lead to lower enthalpic stability for this system. This could be the reason why the INP-2022 system converts water to ice at moderately low temperatures. Again, TmAFP will likely have the least efficiency for the same.

The dynamic properties of water near AFPs and INP-2022 have also been characterized in this study. For this purpose, the mean square displacement (MSD) values have been measured for the surface water molecules near the three systems and are shown in Fig. 2(e). From the figure, a higher MSD value can be observed for TmAFP while the value near INP-2022 is the lowest. Furthermore, we have measured the mean residence time (*τ*) of water molecules which are H-bonded to threonine hydroxyl groups. For this purpose, we start with the water molecules that remain within 3.5 Å of threonine hydroxyl groups and have a donor–acceptor angle within 35°. Then we measured the time taken for these water molecules to exit the first solvation shell from the threonine residues by calculating a correlation function, *C*(*t*). The other details of the residence time calculation are given in the Methods section. This way, we have calculated the *τ* for water near the IBS of SbwAFP, TmAFP and INP-2022. The correlation function with respect to time is shown in Fig. 2(f) for all the systems. From the figure, we see that the correlation function decays most rapidly near the IBS of TmAFP while the decay is slowest near INP-2022. From the exponential decay of *C*(*t*), the value for *τ* can be calculated through fitting the data using a bi-exponential function. From the fittings, the value of *τ* can be obtained for which the equations are given in the Methods section. This way, the value for *τ* was found to be 62.1, 46 and 70.5 ps for SbwAFP, TmAFP and INP-2022, respectively. Therefore, the dynamics of water near the IBS of different systems show a direct correlation with their entropy patterns. A previous study from our group also found a similar pattern for the structure and dynamics of water near INP-2022.^25^ Hence, our analysis finds a clear distinction in the properties of water near INP-2022 compared to AFPs which explains why the AFPs have such low efficiencies for ice-nucleation.

A natural question that arises from our results is whether the water properties observed near the truncated INP-2022 system are preserved near larger INP surfaces. To address this, we performed an additional 10 ns simulation on a longer INP segment consisting of 16 tandem repeats (residues 830–1089), effectively doubling the size of the IBS. The simulation conditions were kept the same as those in the other simulations, and frames were saved every 0.1 ps. From this trajectory, we first calculated the average number of H-bonds per water molecule that are directly H-bonded to the hydroxyl groups of IBS threonine residues, using the same protocol as in Fig. 2(b)–(d). These values, shown in Fig. S9(a) in the ESI,† follow a similar pattern to those in Fig. 2(d), with several water molecules exhibiting significantly fewer hydrogen bonds compared to bulk water. To further examine the water dynamics near the larger INP system, we also computed the correlation function, *C*(*t*), for water molecules H-bonded to IBS threonine residues. The *C*(*t*) *versus* time plots for both the extended INP system and the original INP-2022 system are presented in Fig. S7(b) in the ESI.† In this case as well, a comparable decay pattern is observed, with both systems showing similar relaxation times. These results suggest that, despite using a truncated INP segment in our main analysis, the key water properties identified are likely to remain qualitatively consistent across larger INP surfaces. In the next sections, we investigate the reasons behind the entropy patterns observed for the systems studied here.

### Rotational motions of IBS threonine residues and rotational entropy are correlated

**Fig. 3:**
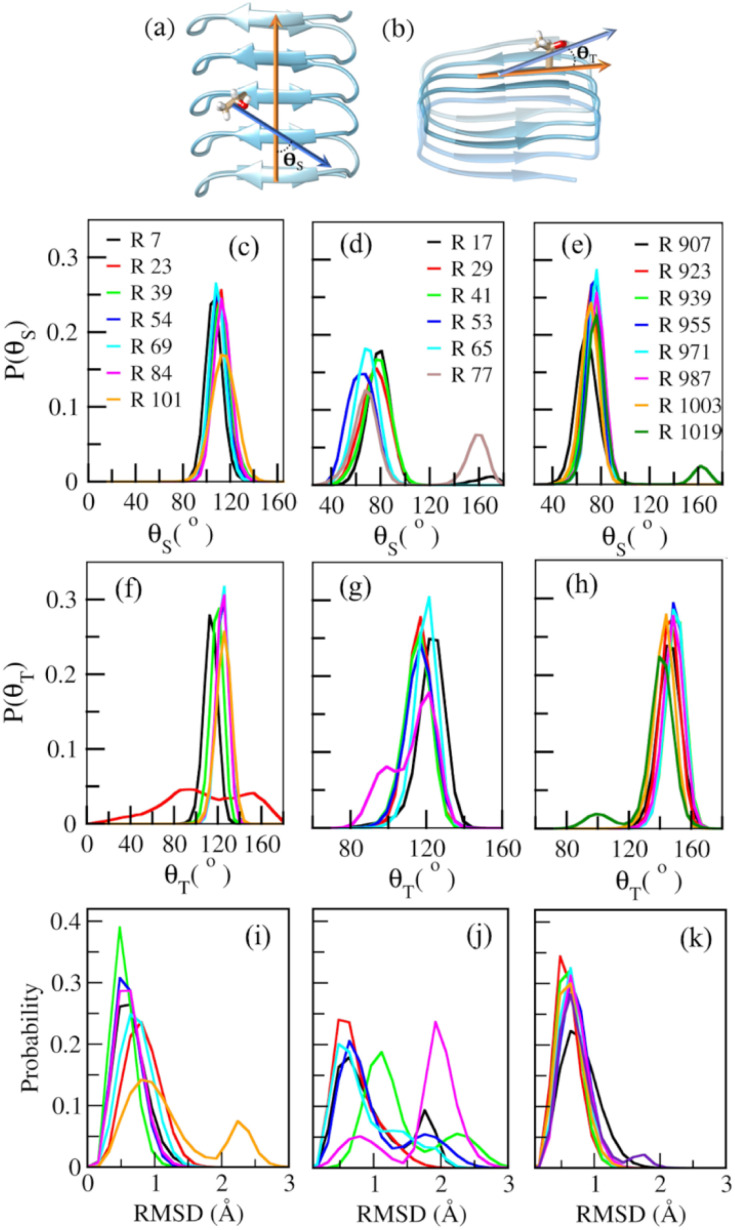
The vectors that define the side angle, *θ*_S_ (a) and the top angle, *θ*_T_ (b) for the threonine residues on the IBS. The orange color vectors are defined by joining the C_α_ atoms from two residues located between two IBS threonine residues for *θ*_S_ as shown in (a) and by joining C_α_ atoms of adjacent threonine residues for *θ*_T_ as shown in (b). The blue vectors in both cases have been defined by connecting the C_β_ atom and hydroxyl oxygen atom of threonine residues for which the angles will be calculated. The panels (c)–(e) present the distribution of *θ*_S_ and panels (f)–(h) present the distribution of *θ*_T_ for SbwAFP ((c) and (f)), TmAFP ((d) and (g)) and INP-2022 ((e) and (h)). The letter R represents the residue numbers in each case. Distribution of root mean square deviation (RMSD) of threonine side chains from the IBS of (i) SbwAFP, (j) TmAFP, and (k) INP-2022.

Since we observe a difference in *TS*_Rot_ values for water molecules directly H-bonded to the hydroxyl group of threonine residues on the IBS, it is interesting to check whether the rotational motion of the threonine side chains correlates with the entropy pattern or not. Therefore, to check how differently threonine side chains on the IBS of AFPs and INPs rotate in solution, angular motions of hydroxyl oxygen with respect to the C_β_-atom of individual threonine residues have been investigated by measuring the two angles shown in Fig. 3(a) and (b). The first angle has been measured by determining the angle made between a vector constructed by connecting the C_α_ atoms of x-residue from the TxT patch on the first and last β-sheet on the IBS (shown using an orange arrow in Fig. 3(a)) and the vector constructed by connecting the C_β_ atom and hydroxyl oxygen (shown using a blue arrow in Fig. 3(a)) on the threonine residue for which the calculation will be done. We term this angle side angle, *θ*_S_. The other angle has been constructed using the same vector shown in blue color in Fig. 3(a) (light blue vector shown in Fig. 3(b)) and another vector constructed through connection of C_α_ atoms of two neighboring threonine residues on the same β-sheet at the IBS, shown in the orange color vector in Fig. 3(b). This angle has been termed the top angle, *θ*_T_. Fig. 3(c)–(h) show the normalized distribution of *θ*_S_ (Fig. 3(c)–(e)) and *θ*_T_ (Fig. 3(f)–(h)) for the left side threonine ladders of SbwAFP, TmAFP, and INP-2022. The distributions for the right side ladder for these systems are shown in Fig. S10 in the ESI.† For SbwAFP's threonine ladder, shown in Fig. 3(c) and (f), we see that nearly all the residues appear to show peaks at the same position for both *θ*_S_ (see Fig. 3(c)) and *θ*_T_ (Fig. 3(i)), except for only one angle for *θ*_T_. For the TmAFP threonine residues shown in Fig. 3(d) and (g) for *θ*_S_ and *θ*_T_, respectively, we find that residues show a broader distribution for *θ*_S_, as evident from lower peak heights. The distribution highlights the flexibility of threonine residues within the IBS of TmAFP, which is likely to affect the *TS*_Rot_ of water molecules H-bonded to threonine hydroxyl groups. For the rotation of INP-2022 threonine residues shown in Fig. 3(e) and (h) for *θ*_S_ and *θ*_T_, respectively, we see mostly sharp peaks except for one terminal residue. For the right-side ladder shown in Fig. S10 in the ESI,† the peaks have slightly smaller heights for both *θ*_S_ and *θ*_T_ in all cases indicating slightly broader distribution and more flexibility for these residues. Overall, the plots indicate that threonine residues from INP-2022 show the least rotational movement among the three systems considered. SbwAFP also shows a similar trend, at least for one of its threonine ladders, which explains why we see such low *TS*_Rot_ values near these residues. This also explains why TmAFP shows bulk water like *TS*_Rot_ values at 273 K and hence will be unlikely be effective for ice nucleation at moderately low temperatures.

A closer inspection of the IBS reveals the subtle difference in the structure of TmAFP from SbwAFP and INP-2022 which is likely to create a difference in the rotational motion of IBS threonine residues. Calculation of average distances between C_α_ atoms from two threonine residues separated by one other residue on the same β-sheet of the IBS reveals that during the simulation, the threonine residues in the adjacent layers are separated by 7.1 Å in TmAFP, while the distance is 6.9 Å for both SbwAFP and INP-2022. Although the difference in the distances seems small, the antifreeze activity has been reported to depend on this particular distance.^54^ Therefore, the slightly larger space between the threonine residues is likely to make them more flexible for TmAFP compared to others.

This assumption has been verified through measurement of root mean square deviation (RMSD) of non-hydrogen atoms of threonine side chains for SbwAFP, TmAFP and INP-2022 and the values are shown in Fig. 3(i)–(k), respectively. From the figure, it is evident that fluctuations in TmAFP are different compared to others. For SbwAFP, shown in Fig. 3(i), threonine residues towards the center of the structure have lower RMSD distribution compared to the terminal residues. For INP-2022, the RMSDs are even more similar for all the residues (Fig. 3(k)). However, the RMSDs of threonine residues on TmAFP have very different distributions, as seen from Fig. 3(j). We claim from this evidence that such fluctuations will govern ice nucleation efficiency of the IBS. The lower the fluctuations, the more effective the ice nucleation.

Since the IBSs of both AFPs and INPs are capable of binding to the growing ice surface, we checked how the fluctuations vary for the threonine residues during the ice binding event. We hypothesize that in spite of the difference in fluctuations between TmAFP and the other systems studied here, the threonine residues behave in a similar manner for all of them during an ice binding event. To test this hypothesis, we have carried out ice growth simulation in the presence of AFPs and INP-2022. Since ice nucleation is a rare event and can take a significant amount of simulation time without any additional bias, we have followed the protocol recently used in another study from our group.^50^ The other details are given in the Methods section. Here we have chosen the temperature to be 265 K instead of 273 K to facilitate the ice growth in the system. During the simulation, the water molecules next to the ice slab convert to ice, eventually reaching the IBS much before 50 ns. From the trajectories, we have calculated the distribution of *θ*_S_ and *θ*_T_ for IBS threonine residues at time intervals between 0 and 5 ns, 15–20 ns, 25–30 ns and 45–50 ns to check how the angle distributions evolve in the vicinity of the ice growing surface. The initial and final stages of the simulations are shown in Fig. S11(a) and (c)† for SbwAFP, TmAFP, and INP-2022. Fig. S11–S14 in the ESI† show the distribution of *θ*_S_ and *θ*_T_ at the above-mentioned time intervals for two threonine ladders from SbwAFP, TmAFP and INP-2022.

From the figures, it emerges that for all cases, although the hydroxyl groups of threonine residues are not fully aligned with each other initially on the same ladder at 265 K, the threonine residues start to become more ordered as the growing ice phase comes near the IBS. This can be observed for all the systems with a slight increase in peak height at the last phase of the simulation. These observations are more prominent for the TmAFP system shown in the middle panel of Fig. S11–S14 in the ESI† with the peak heights increasing from 0–5 ns distribution to the one between 45 and 50 ns. Interestingly, we find that even when the IBS is bound to ice, the distribution of *θ*_S_ can vary for different threonine residues on the same IBS. Furthermore, the distribution observed between the 45 and 50 ns time interval is similar for all the systems. These patterns indicate that during an ice binding event, the IBS from all the systems behave in a similar manner. Therefore, the key difference between AFPs and INPs comes from the way they modulate the water properties in their vicinity before ice formation. The different extents of fluctuations associated with the IBS of different systems are responsible for modulating the water properties as has been observed for different materials^47,55^ as well. In the next section, we investigate factors which can contribute towards the fluctuations of IBS residues and consequently the ice nucleation efficiency of INPs.

### Comparison of water entropy between two INP models

**Fig. 4:**
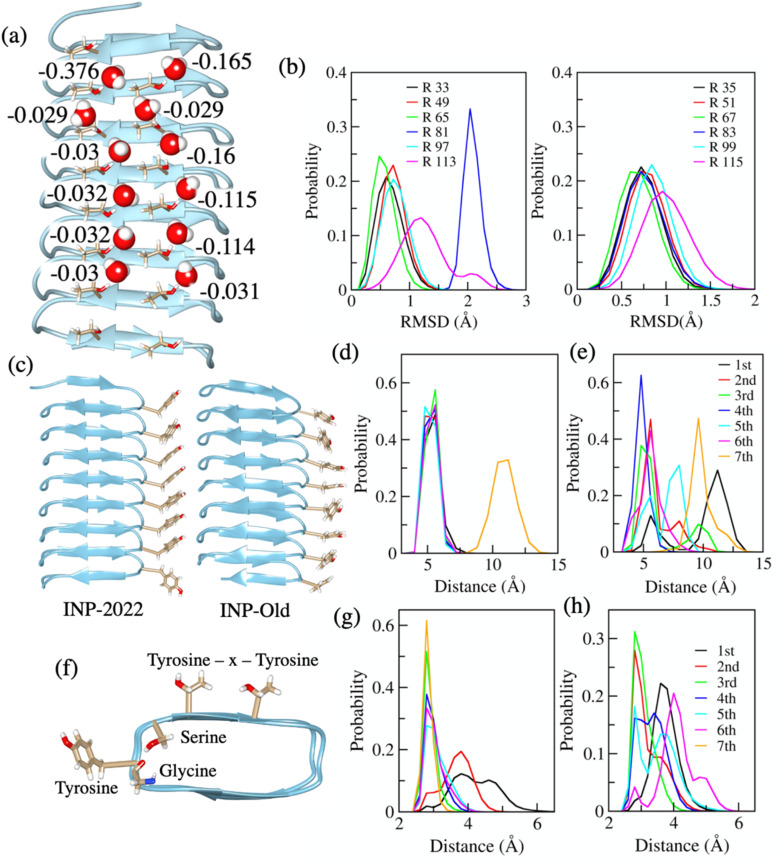
The *TS*_Rot_ values of water molecules H-bonded to threonine residues on the IBS of the INP-old system (a). (b) RMSD distribution of threonine side chains from the IBS of the INP-old system. The left and right panels show the distributions from two threonine ladders on the IBS. The letter R represents the residue numbers for each plot. (c) The positions of tyrosine residues (shown with stick model) on the conserved loop of INP-2022 (left) and INP-old (right) systems. (d) and (e) The distribution of distances between C_β_ atoms of adjacent tyrosine residues from INP-2022 and INP-old, respectively. The numbering 1st to 7th denotes the tyrosine residues from which the distances have been calculated in each case. (f) Positions of serine, tyrosine and glycine residues on the conserved loop region of INPs. The position of the TxT repeats on the IBS is also shown for clarity. (g) and (h) The distribution of distances between carbonyl oxygen of glycine and hydroxyl oxygen of serine residues on the conserved loops from INP-2022 and INP-old, respectively. The numbering 1st to 7th is the loop numbers on these systems from which the distances have been calculated.

To identify the structural features that control the fluctuations of IBS threonine residues, we compare the entropy pattern of INP-2022 with that of another segment of the INP model obtained from Alphafold-1. The other INP system, referred to here as INP-old, has the same number of residues as INP-2022. The INP-old structure has been simulated in the same way as the other systems and then water entropy has been calculated for water near the IBS of INP-old. In Fig. 4(a), the *TS*_Rot_ values of water molecules H-bonded to threonine hydroxyl groups are shown. From the figure, we see that the values of *TS*_Rot_ here are found to be very much similar to those of bulk water unlike the case of INP-2022. This pattern points to the fact that the presence of a continuous threonine ladder may not be a sufficient condition for having low entropy water in the vicinity. To investigate the origin of the different entropy pattern between INP-old and INP-2022, we look at the structural difference between the two systems.

As discussed earlier, the fluctuations for threonine residues are key in governing the values of *TS*_Rot_ for water H-bonded to threonine hydroxyl groups. Therefore, we look at the RMSD of threonine non-hydrogen atoms from the side chain first to determine if the residues exhibit increased fluctuations. The RMSD plot is shown in Fig. 4(b) for the residues from the two ladders. The figure illustrates that, for INP-2022, only the terminal residues show high RMSD values for a few frames, whereas the middle residue on the INP-old structure consistently demonstrates a high RMSD value. The plots indicate more fluctuation for INP-old threonine residues, which is likely causing the water molecule to behave like bulk water.

Upon investigation of the equilibrated systems for INP-old and INP-2022, which were used for entropy calculation, we find a significant difference between the conserved loop region next to the IBS for the two systems. For the loops with the ala–gly–tyr–gly–ser sequence, we observed that the aromatic rings on tyrosine side chains remain stacked with each other in the INP-2022 structure. However, for the INP-old system, such stacking is absent as shown in Fig. 4(c). Here the structures shown are the ones that have been used for simulations with which the water entropy has been calculated. The lack of stacking has been verified through measurement of distances between the C_β_ atoms from the aromatic ring on tyrosine residues which are placed next to each other. The distributions of these distances are shown in Fig. 4(d) and (e) for INP-2022 and INP-old, respectively. From the plots, it can be said that the peaks of the distributions appear at the same position with similar heights for INP-2022, except for one terminal residue, which is only possible when the side chains remain stacked with each other during the simulation. However, the distributions appear at different positions in the case of INP-old, an indication of lack of stacking. Furthermore, we see that for the INP-2022 structure, the serine hydroxyl group makes a H-bond with the glycine carbonyl oxygen on the backbone as shown in Fig. 4(f). To check the presence of this H-bond between INP-2022 and INP-old, we have measured the distance distribution for the serine hydroxyl oxygen and glycine carbonyl oxygen for all the loops. The distributions are shown in Fig. 4(g) and (h) for INP-2022 and INP-old systems, respectively. From the plots, it can be observed that while the INP-2022 structure forms stable H-bonds between these residues as evident from sharp distributions for most of the residues, the distributions are broader in the case of INP-old, indicating the presence of larger fluctuations. Therefore, from the distribution of distances between interloop tyrosine residues and between intra-loop serine–glycine residues, it can be said that the loops on the INP-old structure next to IBS threonine residues are more dynamic in nature compared to the INP-2022 system. We hypothesize that these dynamic loop regions near the IBS affect the threonine residues to fluctuate more and consequently cause less impact on the water entropy behavior.

To test this hypothesis, we have constructed a structure with the sequence of the INP-old system where the tyrosine residues have been placed in a manner that they remain stacked. We call this system INP-old-rigid. After arranging the tyrosine residues in a stacked manner, no restraints have been imposed on the system and the system has been simulated in a similar manner to the other systems. A final 200 ns long trajectory has been generated for *TS*_Rot_ calculations near the IBS of this system. Interestingly, we found that on this system, the tyrosine residues remained stacked even at the end of 200 ns long simulation without any additional bias. Therefore, we can expect the loop region for this system to be more rigid compared to the structure in which the tyrosine residues do not remain stacked. To verify how the loop rigidity affects the threonine residues' fluctuations in this new INP-old structure, we have again calculated the RMSD of IBS threonine side chain heavy atoms. The plot is shown in Fig. S15 in the ESI.† The figure clearly shows that compared to the fluctuations shown in Fig. 4(b), the threonine fluctuations on the new structure of INP-old are significantly less. This is true for both threonine ladders and for all the residues. Next, we calculated the *TS*_Rot_ for water molecules that remain H-bonded to threonine hydroxyl groups on the IBS. The entropy values for these water molecules are shown in Fig. S16 in the ESI.† From the values, we can see that with lowering of fluctuations for threonine side chains, the entropy values also show lower values compared to the other structure of INP-old where the tyrosine residues do not remain stacked. The lower values compared to bulk water observed here clearly indicate that the continuity of the water-organizing motif is not the only criterion that can determine the ice nucleating efficiency of an IBS as stated earlier.^39^ The fluctuations in these water organizing motifs also play a significant role in determining the ice nucleation temperatures on these surfaces. Therefore, future attempts at designing INPs should also involve efforts to make the structure rigid through interactions within the structure for better efficiency.

## Conclusions

The significance of ice formation in nature makes it imperative to better understand the mode of operation of ice nucleating agents (INAs). Ice nucleating proteins are unique systems to study due to their similar structural characteristics to AFPs having an opposite function. Hence, identifying the subtle differences between these classes of proteins is crucial for future designs of INAs with better efficiencies. In this study, we have progressed in this direction through investigating the effect of biological anti-freeze or ice-nucleating proteins on their surrounding water molecules. Using a method that calculates the single water entropy, we characterized the water molecules in the vicinity of threonine residues on the IBS of these proteins in terms of rotational entropy of water. Formation of ice at lower temperatures would likely result in lowering of entropy. Hence, the presence of water molecules having lower entropy values should decrease the entropic barrier and enhance the efficiency of ice nucleation near the IBS of AFPs and INPs. Our calculations indicate that among the two AFPs, the SbwAFP system reduces the *TS*_Rot_ for several water molecules near the threonine residues on the IBS. However, for the other AFP named TmAFP, the reduction in entropy is found to be much less compared to the SbwAFP system. The entropy patterns have also been found to be in accordance with their thermal hysteresis data where SbwAFP is known to be a better antifreeze agent than TmAFP. Hence, it is likely that improvement in antifreeze efficiency of AFPs depends on the extent of their water ordering ability.

For the recent INP structure obtained through Alphafold v2.0, it was found that the number of water molecules having significantly low *TS*_Rot_ is considerably higher than the AFPs. The results implied that such lowering of entropy enables the IBS of INPs to overcome the entropic barrier of ice nucleation, resulting in conversion of liquid water into ice under moderate supercooling conditions. Using our method to probe properties of individual water molecules, we have also investigated the tetrahedrality, O–O–O angle distribution, average number of H-bonds and some of the dynamic properties of water molecules in the vicinity of threonine residues of the IBS. The O–O–O angle distribution and conditional tetrahedral parameter showed the water molecules near the INP-2022 system to have the highest ordering while they possess fewer H-bonds per water. The TmAFP system showed the highest number of H-bonds for water molecules with the least tetrahedrality. These patterns indicate that water near TmAFP is similar to bulk water. The dynamics of these water molecules near these systems also showed a similar pattern. The water near the INP-2022 system showed the slowest dynamics while the water near TmAFP has the least mean residence time and highest diffusion. Such differences make the INPs unique in terms of their ability to nucleate ice. Our investigation finds that the water entropy and structural and dynamic patterns are all dependent on the fluctuations of side chains of threonine residues on the IBS. A slight difference in the distances between threonine residues on the IBS of TmAFP compared to SbwAFP or INP-2022 makes the side chain of this system fluctuate more than the others. These fluctuations have a direct impact on the ordering of its neighboring water molecules. We also investigated the impact of side chain fluctuations on the ice binding process on the IBS of AFPs and INPs. Our analysis finds that during the ice binding process, the AFPs and INPs show similar side chain fluctuations at the same temperature. This indicates that while the ice nucleation efficiency for these proteins can differ at moderately low temperatures, their ice binding ability will be very similar to each other.

The significance of structural fluctuations gets even more prominent when we have compared the entropy patterns for INP structures from two different models. In the INP-2022 system, the loop residues next to IBS threonine residues remained rigid during the simulation. This is due to π–π stacking between the tyrosine side chain and an intra-loop H-bond between serine and glycine carbonyl oxygen. No stacking was found during the simulation of the INP-old system. This resulted in a more fluctuating loop region for the system. Interestingly, we found that the IBS threonine residues on the INP-old system also showed more fluctuations, which was found to reduce when the loop was rigidified. These observations point to the fact that not only the large surface area for the IBS or a continuous patch of threonine is sufficient to make a system an effective ice nucleator. The system will require a certain extent of rigidity that collectively imparts ice like character to the water molecules in the solvation shell so that ice nucleation can take place under moderate supercooling conditions.

Overall, the results from our studies will be helpful in developing better ice nucleating agents in the future. Furthermore, neural network-based models are often used to predict the ice nucleation efficiency of an IBS. From our study, we suggest that structural fluctuations could also be a crucial parameter to include in these methods. A rigid surface would likely slow down the dynamics of solvation shell water more effectively than a fluctuating surface. Also, the ordering of water required to nucleate ice will be more pronounced near rigid surfaces. The proof of these concepts will only come through future studies on more such systems.

## Methods

### Molecular dynamics simulations

The molecular dynamics (MD) simulations were performed using the GROMACS 2018.3 ^56,57^ package with the AMBER99SB-ILDN force field.^58,59^ A TIP5P water model has been used for all the cases. The proteins mentioned here have been inserted in a triclinic box and the systems were solvated with water molecules. The required number of sodium or chloride ions was added to the systems for neutralization. The final systems corresponding to SbwAFP, TmAFP, INP-2022 and INP-old contained 12 381, 9244, 15 857 and 14 739 water molecules, respectively. The systems were then first energy minimized using the steepest descent method.^60^ The minimized systems were then simulated for 5 ns under NVT conditions, followed by another 5 ns simulation under NPT conditions where backbone atoms of the protein were restrained with a force constant of 1000 kJ mol^−1^ nm^−1^. Nosé–Hoover thermostat^61,62^ was used to maintain the temperature at 273 K with a coupling constant of 0.5 ps. The pressure was maintained at 1 atm using Berendsen barostat^63^ with a coupling constant of 0.5 ps. The particle mesh Ewald^64^ summation method was used to calculate long range electrostatic interactions with a cut-off of 1.0 nm and a grid spacing of 0.16 nm. A 1.0 nm cut-off was used for the van der Waals (vdW) interactions. The equilibrated simulation box had dimensions of 7.01 × 6.66 × 8.34 nm^3^, 7.92 × 6.18 × 5.87 nm^3^, 8.59 × 6.83 × 8.42 nm^3^ and 7.96 × 7.48 × 7.75 nm^3^ for SbwAFP, TmAFP, INP-2022 and INP-old systems, respectively. Using these equilibrated systems, a final trajectory of 200 ns under NPT conditions has been generated for each system with which the entropy calculations have been performed. The frames have been saved at every 0.1 ps for these trajectories.

### Rotational entropy calculation

The method used for rotational entropy calculation has been given in ref. 43. Here, for our calculations, we first use the method named permutation reduction^65,66^ developed by Grubmüller and co-workers, which keeps the identity of each water molecule with respect to a reference configuration. This way, the water molecule at a particular position moves in a localized region only. For calculation of rotational entropy of individual water molecules, distribution of two angles has been considered. The first angle, *θ*, is the angle between the water oxygen to the solute vector and the dipole vector. The other angle, *χ*, is the angle between the normal to the plane defined using the solute-oxygen vector and the dipole vector and the H–H vector of the water. With the angular distribution *p*(*θ*, *χ*), the rotational entropy has been calculated using the following equation:

1 *S*_Rot_ = −*k*_B_∫*p*(*θ*, *χ*)*c* ln{*p*(*θ*, *χ*)*c*} sin *θ* d*θ* d*χ*

### Ice growth simulations

Since ice growth can involve significantly long timescales due to high entropic barriers, we employed a method in which ice growth near the IBS can be observed within a short period. Therefore, an ice slab is first generated using the Genice^67^ software package. A few layers of such ice are then taken, and the protein is placed at about 1 nm distance from the ice surface. The rest of the simulation box is filled with water molecules. To prevent ice growth from both sides of ice, few water molecules on the opposite side of proteins have been position restrained using a force of 1000 kJ mol^−1^ nm^2^. The system has been equilibrated at 265 K temperature and 1 bar pressure for 10 ns in the same manner as the other systems from which entropy values have been calculated. After the equilibration, 50 ns long simulation at the same temperature and pressure has been carried out to observe the ice binding event on the AFPs and INP-2022. Visual inspection indicated that the ice formation took place near the protein before 50 ns.

### Mean residence time calculation

For the calculation of mean residence time of water H-bonded to threonine residues on the IBS, three residues from the middle regions of the threonine ladder on the IBS have been taken from all the systems. The method used for the calculation has been based on the stable state picture (SSP) approach of chemical reactions^68^ previously used for calculation of residence time of water in the solvation shells of halide ions^69^ and DNA base pairs.^70^ Here, for our purpose, we have defined the reactant state (r) for a water molecule when it remains H-bonded to the threonine hydroxyl group at time *t* = 0. The water goes to the product state (p) when the H-bonded water goes beyond 0.53 nm from the IBS at time *t* = *τ*, which is the residence time. The distance cut-off chosen here has been based on the radial distribution function (RDF) calculated for water oxygen atoms with the IBS of SbwAFP taken as a reference. In this method, the transient recrossing by the water molecules from the reactant state is taken into account by choosing the product state properly. After defining the reactant and product states, we calculate the following correlation function, *C*(*t*), using the probability of the reactant state, *p*_R_ and *p*_P_, as:

2 *C*(*t*) = 1−〈*p*_R_(0)*p*_P_(*t*)〉.

After obtaining *C*(*t*), the function has been fitted with the following bi-exponential function:3
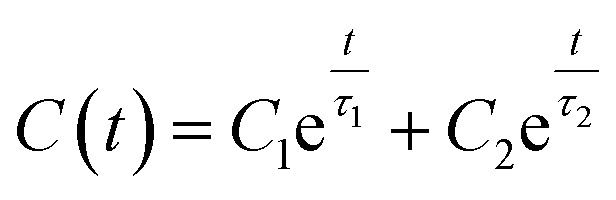
where *C*_1_ and *C*_2_ are the coefficients and *τ*_1_ and *τ*_2_ are two relaxation timescales. From this, we can obtain the mean residence time using the following relation:4
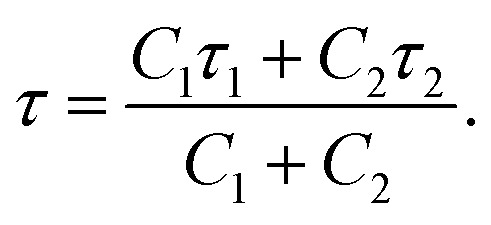


## Author contributions

D. S., R. A., A. M. and B. J. designed research; R. A. and D. S. performed the simulations; D. S. and R. A. performed the analysis; D. S., R. A., A. M. and B. J. wrote the manuscript.

## Conflicts of interest

There are no conflicts to declare.

## Supplementary Material

SC-016-D4SC08383K-s001

## Data Availability

The data supporting this article have been included in the ESI.†
