# Correction: Water entropy at the threonine-rich surface of antifreeze and ice-nucleating proteins: small changes make a big difference

**DOI:** 10.1039/d5sc90146d

**Published:** 2025-07-08

**Authors:** Debasis Saha, Rahul Aich, Arnab Mukherjee, Biman Jana

**Affiliations:** a College of Integrated Science and Arts, Arizona State University Mesa Arizona 85212 USA; b School of Chemical Sciences, Indian Association for the Cultivation of Science Jadavpur Kolkata 700032 India pcbj@iacs.res.in; c Department of Chemistry, Indian Institute of Science Education and Research Pune 411008 India arnab.mukherjee@iiserpune.ac.in

## Abstract

Correction for ‘Water entropy at the threonine-rich surface of antifreeze and ice-nucleating proteins: small changes make a big difference’ by Debasis Saha *et al.*, *Chem. Sci.*, 2025, **16**, 10771–10784, https://doi.org/10.1039/D4SC08383K.

In the original version of this manuscript, the name of the first author was spelt incorrectly. The intended spelling of the first author, Debasis Saha, is given in this correction.

The Royal Society of Chemistry apologises for these errors and any consequent inconvenience to authors and readers.

