# Supplementary material for: Water entropy at the threonine-rich surface of antifreeze and ice-nucleating proteins: small changes make a big difference
Source: Chem Sci. 2025 May 9;16(24):10771–84. doi: 10.1039/d4sc08383k (PMC12091574; doi:10.1039/d4sc08383k)
Supplement: SC-016-D4SC08383K-s001 [file SC-016-D4SC08383K-s001.pdf]

Supplementary Information (SI) for Chemical Science.
This journal is © The Royal Society of Chemistry 2025

Supporting Information
For

Water Entropy at the Threonine-rich Surface of Antifreeze and Ice-nucleating Proteins: Small Changes make Big Difference

Debasis Saha, Rahul Aich, Arnab Mukherjee* and Biman Jana*

Convergence of $TS_{Rot.}$ values:

To check the convergence of the calculated entropy values, we have calculated the TSRot. values with permuted trajectories of different lengths. For this purpose, we have selected random water molecules near the SbwAFP system and the convergence of water entropy having a wide range of values have been evaluated at the time frames shown in Fig. S1. From the plot, it indicates that the entropy values have converged for all the systems during the 200ns long simulations carried out at 273K.

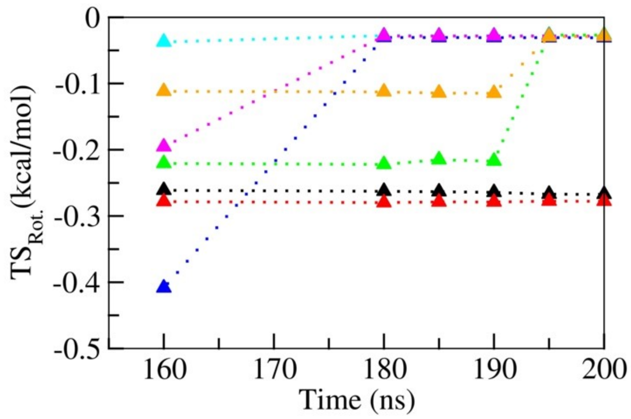

Figure S1 Convergence of TS_{Rot} values for different water molecules shown in different colored triangles from the solvation shell of SbwAFP system. Each of the triangles show a value of entropy obtained from permuted trajectory of the given length. The dotted lines are used only to guide the eyes.

Error estimate for TS_{Rot}:

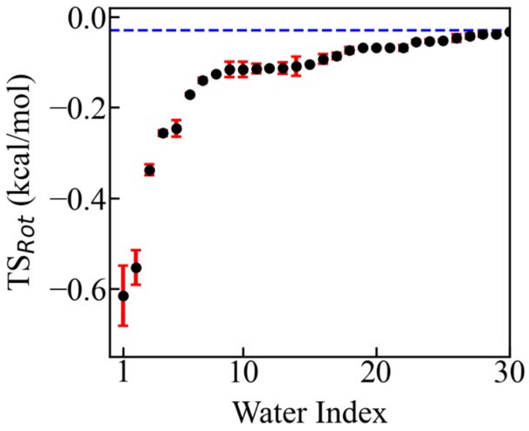

Figure S2 The TS_{Rot} values (black circles) along with their error bars (red) for closest 30 water molecules near the IBS of SbwAFP system.

Entropy of water near threonine residues from a non-AFP surface:

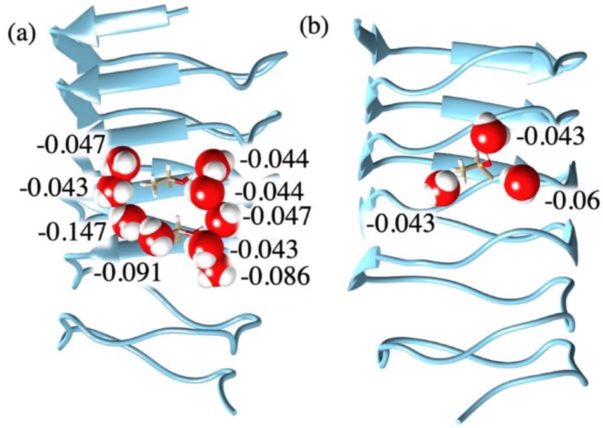

Figure S3 The $TS_{Rot.}$ values of water near three different threonine residues from the surface of a non-AFP. The values correspond to the entropy of each individual water molecules. The units for entropy are in kcal/mol.

Translational Entropy (TS_{Trans}) for Water molecules near Threonine residues at IBS:

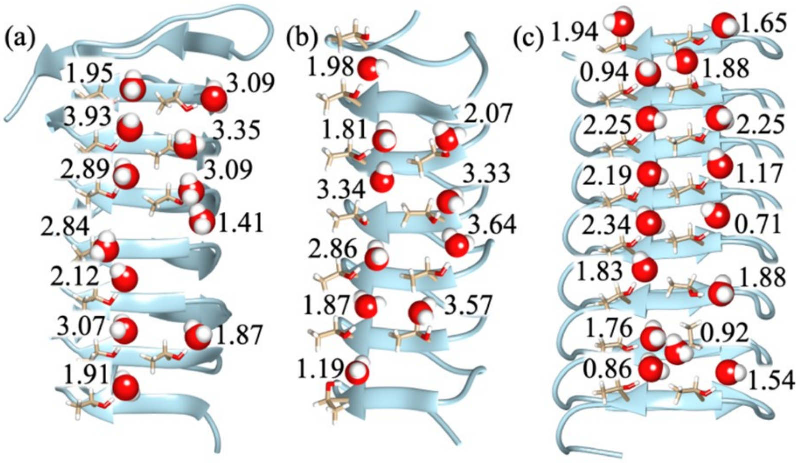

Figure S4 Translational entropy values, TS_{Trans} with anharmonic contribution for individual water molecules H-bonded to threonine hydroxyl groups on the IBS of SbwAFP (a), TmAFP (b) and INP-2022 (c). The units for the values are kcal/mol.

Entropy of water near methyl groups of threonine residues on IBS of TmAFP and INP-2022:

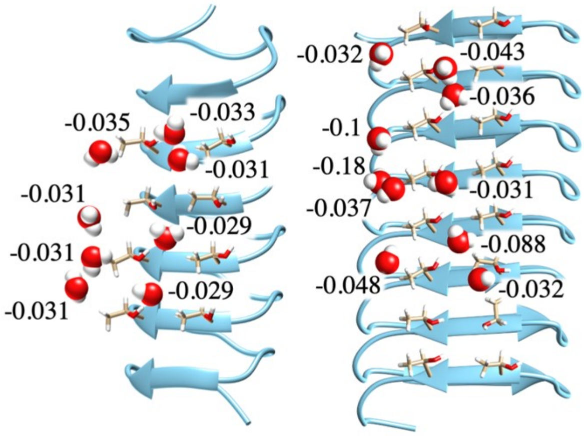

Figure S5 The TS_{Rot} values of water near methyl groups of threonine residues on the IBS of TmAFP (left) and INP-2022 (right) system. The units are in kcal/mol.

Comparison of H-bonds for water near IBS:

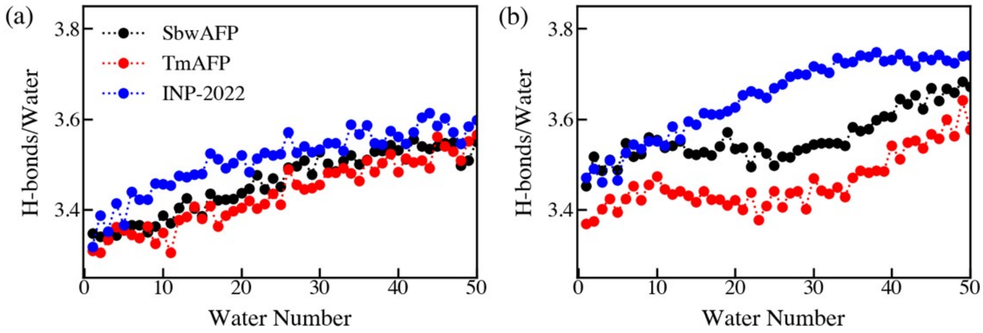

Figure S6 Average number of H-bonds per water for the closest 50 water molecules near IBS of SbwAFP (black dots), TmAFP (red dots) and INP-2022 (blue dots) for water in non-ice state (a) and in ice like state (b). The dotted lines are given to guide the eyes.

Distribution of conditional tetrahedrality of water near IBS of SbwAFP, TmAFP, INP-2022 and from bulk water:

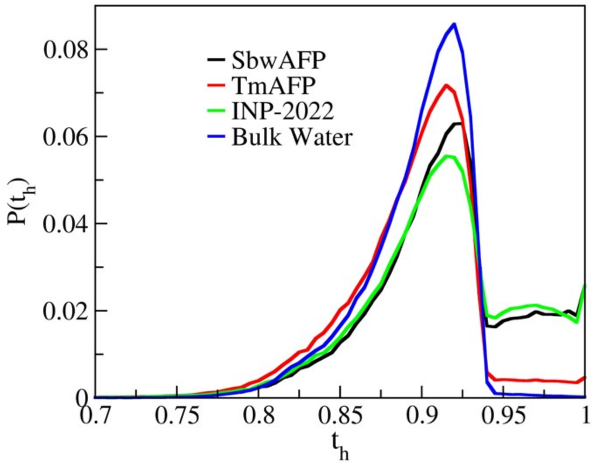

Figure S7 Distribution of t_h values for water near IBS of SbwAFP (black), TmAFP (red), INP-2022 (green) and bulk water (blue) at 273K.

Conditional Tetrahedral Order Parameter for individual water molecules near IBS of SbwAFP, TmAFP and INP-2022:

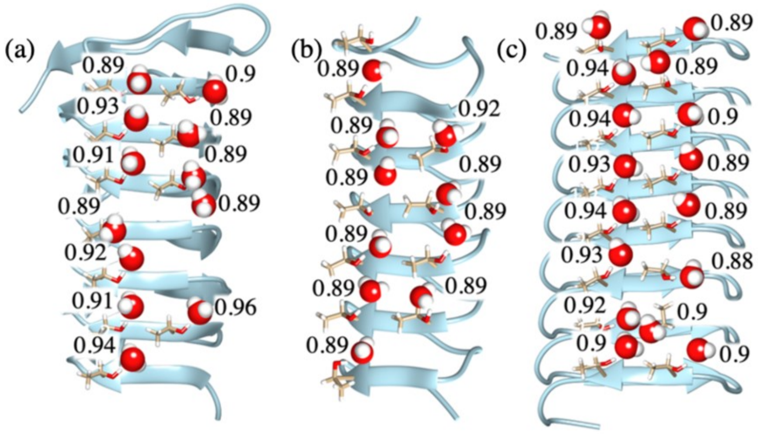

Figure S8 Average number of H-bonds for individual water molecules near the threonine hydroxyl groups on the IBS of SbwAFP (a), TmAFP (b) and INP-2022 (c). The units are in kcal/mol.

Comparison with larger INP segment:

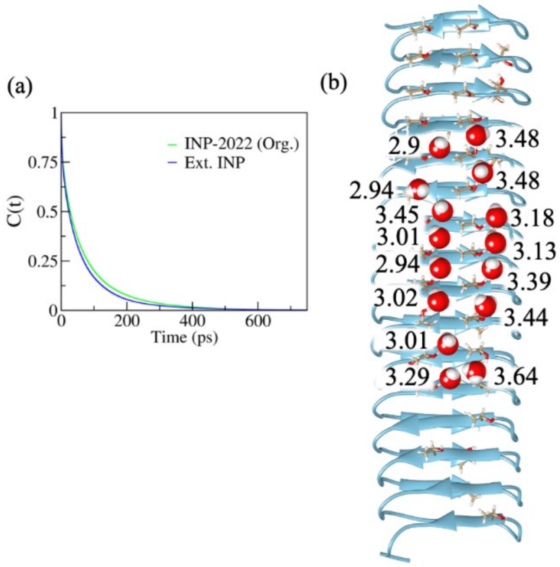

Figure S9 (a) The correlation function, C(t) versus time for the original INP-2022 structure used in the study (green) and the extended INP segment (blue). (b) The average number of H-bonds per water molecules for the water molecules that are H-bonded to the IBS threonine residues in the middle region.

Rotational motions of IBS threonine residues from right side ladders:

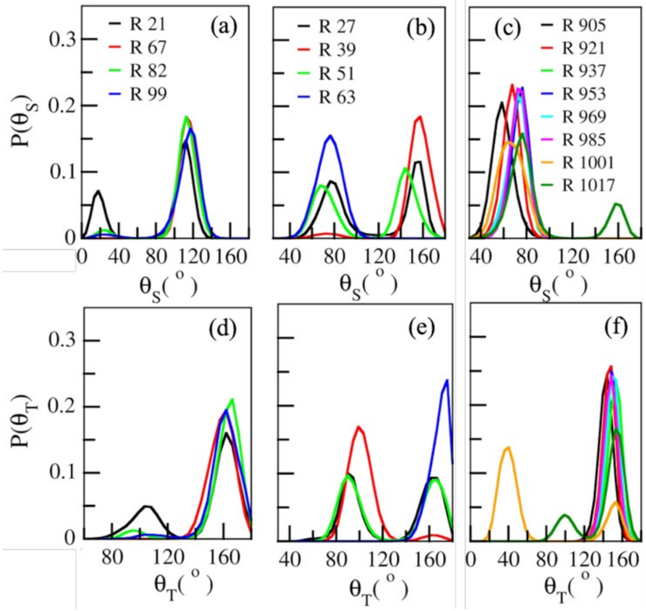

Figure S10 The distribution of θ_S and θ_T for right side threonine ladder on the IBS of SbwAFP (a and d), TmAFP (b and e) and INP-2022 (c and f). The letter R represents the residue numbers in each case.

Distribution θ_S of during the different stages of ice growth simulations for left side threonine ladders on the IBS:

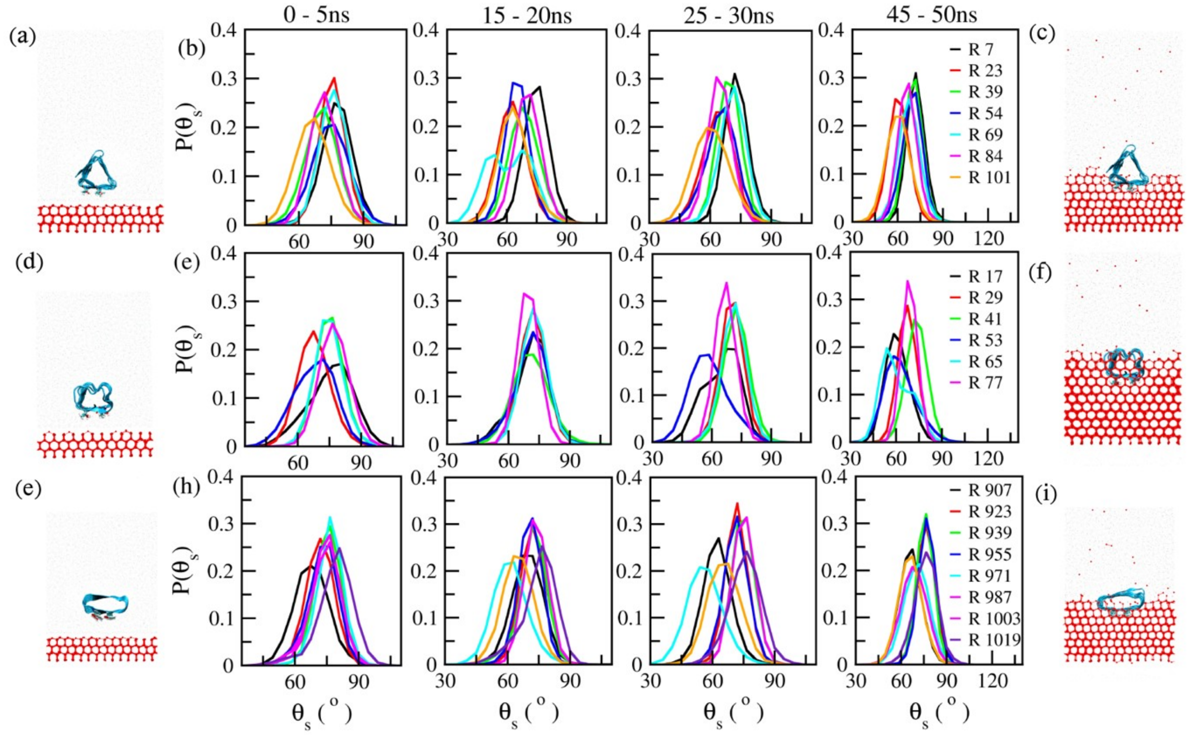

Figure S11 Initial frames at 0th time from ice-growth simulations (left-most column), distributions of θ_S for IBS threonine residues at different time intervals (middle four columns) and final frames after 50 ns simulations (right-most column) for SbwAFP ((a) – (c)), TmAFP ((d) – (f)) and INP-2022 ((e) – (i)) system. For the distributions shown in figures (b), (e) and (h), the time intervals have been mentioned on top of the columns. The letter R represents the residue numbers for each system.

Distribution θ_S of during the different stages of ice growth simulations for right side threonine ladders on the IBS:

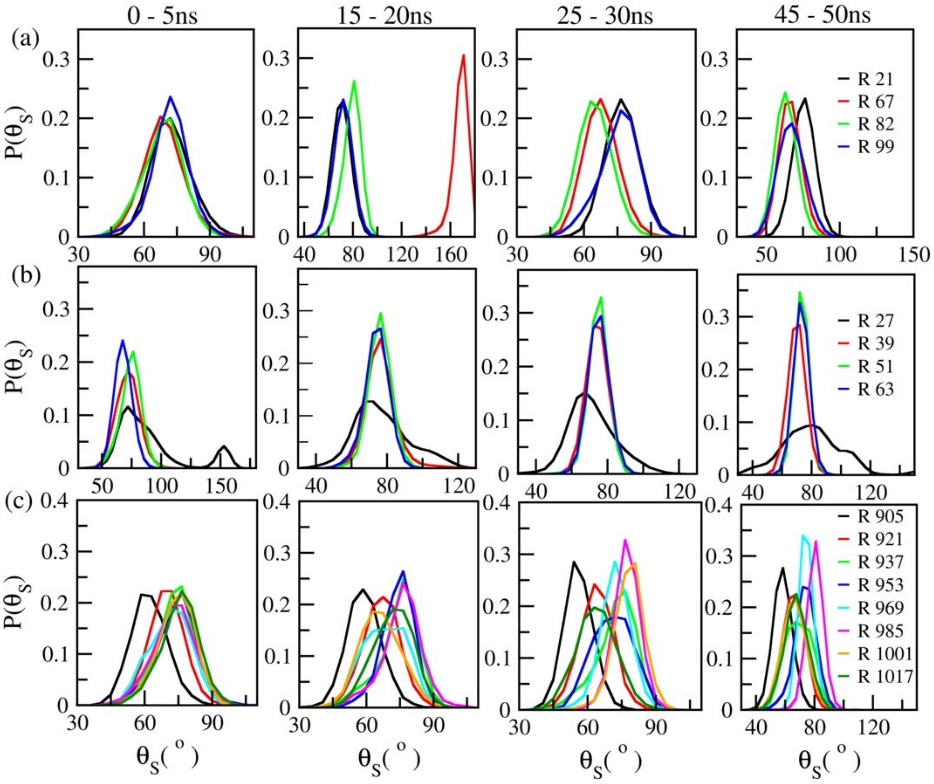

Figure S12 Distributions of θ_S for IBS threonine residues at different time intervals for SbwAFP (a), TmAFP (b) and INP-2022 (c) system. For the distributions shown, the time intervals have been mentioned on top of the columns. The letter R represents the residue numbers for each system.

Distribution θ_T of during the different stages of ice growth simulations for left side threonine ladders on the IBS:

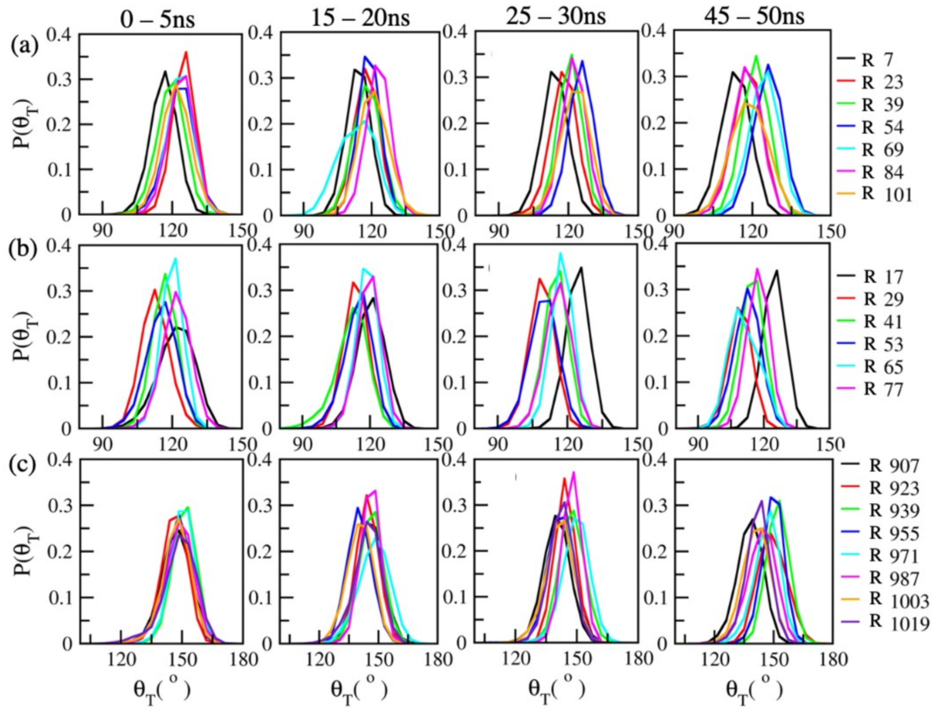

Figure S13 Distributions of θ_T for IBS threonine residues at different time intervals for SbwAFP (a), TmAFP (b) and INP-2022 (c) system. For the distributions shown, the time intervals have been mentioned on top of the columns. The letter R represents the residue numbers for each system.

Distribution θ_T of during the different stages of ice growth simulations for left side threonine ladders on the IBS:

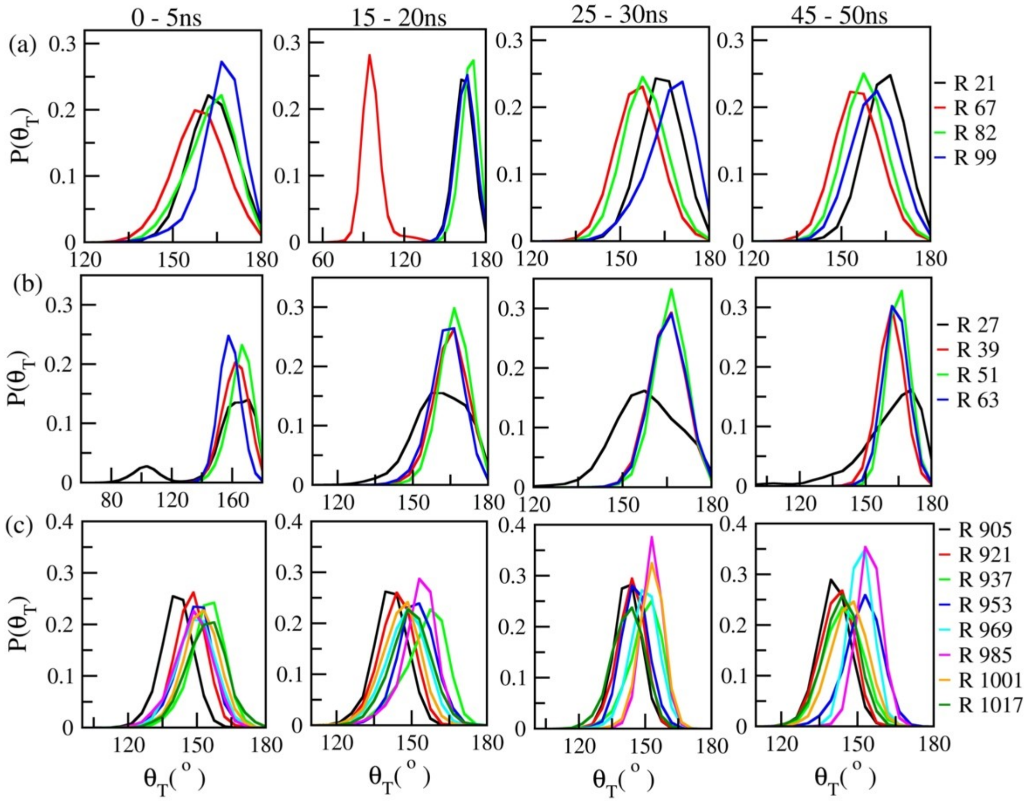

Figure S14 Distributions of θ_T for IBS threonine residues at different time intervals for SbwAFP (a), TmAFP (b) and INP-2022 (c) system. For the distributions shown, the time intervals have been mentioned on top of the columns. The letter R represents the residue numbers for each system.

RMSD distribution for threonine side chains from the IBS of INP-Old-rigid system:

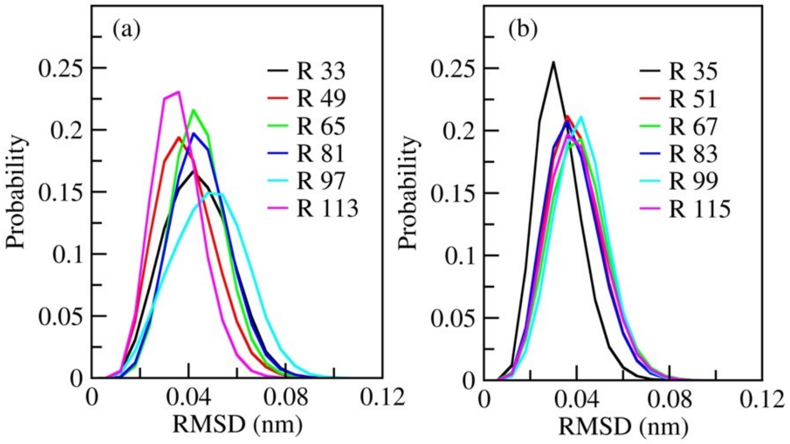

Figure S15 The distributions of RMSD values for threonine residues from the surface of INP-Old-rigid system in which the tyrosine residues on the loop have been stacked on with each other. The R are the residue numbers in the plots.

The TS_{Rot} values for water H-bonded to the threonine residues from the IBS of INP-Old-rigid:

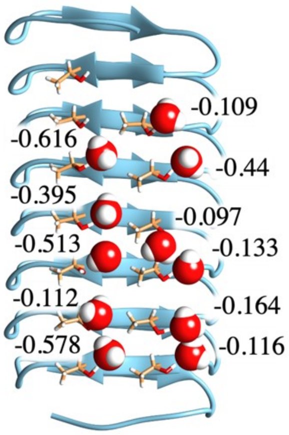

Figure S16 The TS_{Rot} values of water molecules H-bonded to the threonine residues on the IBS of INP-Old-rigid system. The units are in kcal/mol.
